# A microbial transformation using *Bacillus subtilis* B7-S to produce natural vanillin from ferulic acid

**DOI:** 10.1038/srep20400

**Published:** 2016-02-04

**Authors:** Peng Chen, Lei Yan, Zhengrong Wu, Suyue Li, Zhongtian Bai, Xiaojuan Yan, Ningbo Wang, Ning Liang, Hongyu Li

**Affiliations:** 1 School of Pharmacy, Lanzhou University, Donggang West Road No. 199, Lanzhou, 730020, PR China; 2 College of Life Science and Technology, Heilongjiang Bayi Agricultural University, Daqing, 163319, PR China; 3 Gansu Institute of Business and Technology, Yannan Road No. 449, Lanzhou, 730010, PR China; 4 The First Hospital of Lanzhou University, Donggang West Road No. 1, Lanzhou, 730020, PR China

## Abstract

*Bacillus subtilis* strain B7-S screened from18 strains is an aerobic, endospore-forming, model organism of Gram-positive bacteria which is capable to form vanillin during ferulic acid bioconversion. The bioconversion of ferulic acid to vanillin by *Bacillus subtilis* B7-S (*B. subtilis* B7-S) was investigated. Based on our results, the optimum bioconversion conditions for the production of vanillin by *B. subtilis* B7-S can be summarized as follows: temperature 35 °C; initial pH 9.0; inoculum volume 5%; ferulic acid concentration 0.6 g/L; volume of culture medium 20%; and shaking speed 200 r/min. Under these conditions, several repeated small-scale batch experiments showed that the maximum conversion efficiency was 63.30% after 3 h of bioconversion. The vanillin products were confirmed by spectral data achieved from UV–vis, inductively coupled plasma atomic emission spectroscope (ICP-AES) and Fourier transform infrared spectrometer (FT-IR) spectra. Scanning electron microscopy (SEM) and transmission electron spectroscopy (TEM) results confirmed that the cell surface of *B. subtilis* plays a role in the induction of ferulic acid tolerance. These results demonstrate that *B. subtilis* B7-S has the potential for use in vanillin production through bioconversion of ferulic acid.

Vanillin (C_8_H_8_O_3_, 4-hydroxy-3-methoxy-benzaldehyde), one of the most important flavor compounds, is widely used in the food, beverage, cosmetic, pharmaceutical and medical industries^1^,^2^. The increasing market demand necessitates improvements in the yield of vanillin. At present, the market demand for vanillin exceeds 15,000 tons, of which only 2,000 tons are obtained naturally; the remainder is produced by chemical synthesis^3^. However, due to the low yield of natural vanillin production and the slow growth of plant tissues, vanillin production by extraction from plant products is limited^4^,^5^. Furthermore, the productions rate of vanilla cell culture and vanilla bean extract are extremely low and the method is expensive. Nowadays, vanillin is mainly produced chemically via guaiacol and lignin, leading to a cheaper product but of lower quality, environmentally unfriendly production processes and lacks substrate selectivity, which might reduce the process efficiency and increase downstream costs^6^,^7^. Moreover, according to UK and EU legislations, synthesized vanillin are artificial or nature-identical and can thus not be considered for the making of natural product^8^. Thus, the demand of society for consumption of natural flavor additives has been a great effect on the flavor-producing industries, as the majority of today’s flavors need to be natural. Moreover, natural vanillin is sought after in the worldwide market, and its price is 80–267 times higher than that of the synthetic product^7^.

The use of microbial cells and their enzymes as bioconversion in the synthesis of fine chemicals has attracted much attention in the field green chemistry and white biotechnology^1^. As an alternative to traditional methods, bioconversion has been recognized as an effective technique for the production of natural vanillin. Compared to the chemical methods mentioned above, biotransformation of vanillin produces extraordinary increases in productions natural characteristics and health care and is efficient, ecologically safe. There is a growing tendency in the production of vanillin from natural materials by bioconversion, which can then be considered as a natural aroma chemical^9^. Bioconversion can be defined as the catalysis of the conversion of potential precursors (ferulic acid, vanillic acid, eugenol, isoeugenol, phenolic stilbenes, aromatic amino acid, and so on) by isolated enzymes or by microorganisms^3^,^10^,^11^. Enzymatic catalysis cannot match the large-scale synthesis and production of vanillin, and bioconversion using whole microbial cells is therefore considered an important and efficient tool for the mass production of vanillin^7^,^12^,^13^. Recently, research into the production of vanillin by bioconversion has increased due to its important advantages, such as the relatively mild reaction conditions, the high substrate selectivity, and fewer environmental problems^14^,^15^,^16^. Among the above-mentioned vanillin precursors, ferulic acid (4-hydroxy-3-methoxycinnamic acid) is an extremely abundant natural aromatic product in plants, and it occurs as a component of the cell walls in many agriculturally important crops, such as rice wheat, maize and sugar beet^4^,^17^. It has been reported that agro waste containing ferulic acid can be cost-effectively used to produce natural vanillin through microbial conversion rather than conventional chemical reagents^3^.

Although vanillin production via conversion of ferulic acid has been widely reported in various microorganisms, including *Pseudomonas acidovorans*, *Saccharomyces cerevisiae*, *Rhodotorula rubra*, *Streptomyces setonii*, *Bacillus coagulans*, *Streptomyces halstedii*, *Schizophyllum commune*, *Bacillus licheniformis*, *Delftia acidovorans*, *Pseudomonas putida* and *Sphingomonas paucimobilis*^18^,^19^,^20^,^21^,^22^,^23^,^24^. These studies mainly focused on the isolation and identification of transforming strains and the yield of vanillin. Generally, the bioconversion of ferulic acid is a complex process, and many factors may influence the microbial catalysis. However, to the best of our knowledge, there has been no report on the systemic investigation of factors that affect the conversion of ferulic acid. The ability of bacteria to produce natural vanillin from ferulic acid is worthy of study. The fundamental knowledge derived from this study should provide a valuable platform for further investigation into the behavior of bacteria involved in ferulic acid bioconversion and has potential biotechnological applications in natural vanillin production.

In the current study, we screened ferulic acid-degrading bacteria and found a novel strain of *Bacillus subtilis* (*B. subtilis*), labeled as B7-S, capable of biotransformation of ferulic acid to vanillin. The behavior of *B. subtilis* B7 in response to different initial concentrations of ferulic acid was investigated in a series of batch experiments. To industrialize the conversion of ferulic acid by *B. subtilis* B7-S, the small-scale batch experiments was carried out in a 5-L fermenter based on the results of a bioconversion study. In addition, the experimental data on ferulic acid bioconversion were analyzed and simulated by mathematical models.

## Results and Discussion

As shown in Table 1, the production of vanillin by bacteria and actinomyces (>0.010 g/L) is higher than that by fungi (<0.010 g/L). Among the strains screened here, the productions of vanillin by *B. subtillis*, *Enterobacter cloacae*, and *Bacillus coagulans* were the highest, and the productions of vanillin by *Cantharellus cibarius*, *Boletus edulis*, and *Club fungi* were the lowest (<0.003 g/L).

For most of the strains, the molar conversion rate was lower than 10%, except in the case of *B. subtilis* B7 (13.33%). Based on our analysis of the growth conditions, all of the screening strains could grow in the presence of a low concentration of ferulic acid; however, the tolerance concentration of the bacteria was higher than that of the fungal and actinomyces strains. We also carried out experiments with different initial ferulic acid concentrations, and the results showed that the highest tolerant concentration of bacteria and actinomyces were 0.5 g/L, whereas that of the fungi was 0.3 g/L, which is higher than the upper concentration; therefore, the strains could not survive. Thus, we used the two above-mentioned concentrations in our subsequent experiments.

Our results showed that the capacity of the eukaryotic strains for ferulic acid conversion was lower than that of the prokaryotic strains, which might be due to the lower tolerance concentration of the fungi. Ferulic acid conversion by eukaryotes was previously described by Zheng, who found that the molar conversion rate of *Aspergillus niger* SW-33 was as high as 29.35%^25^. Moreover, the results shown in Table 1 revealed that although eukaryotes show strong conversion ability, they also possess a strong vanillin decomposition capacity. Therefore, conversion processes involving eukaryotes will require two-step methodologies.

**Figure 1:**
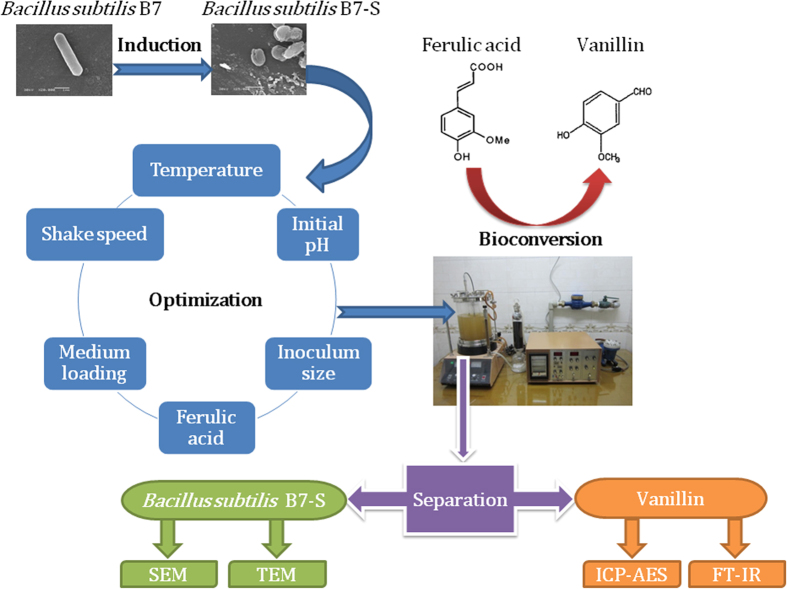
Bioconversion pathway of vanillin by *B. subtilis* B7-S.

On the other hand, the results showed that the conversion rates of bacteria and actinomyces (>10%) were higher than that of fungi (<10%). The production of vanillin by *B. subtillis*, *Enterobacter cloacae*, and *Bacillus coagulans* was higher than the production by other strains. Among the 18 strains tested, the conversion rate of *B. subtillis* B7 was the highest. Meanwhile, we also planned to adopt the most effective one-step method in this experiment. Therefore, we chose *B. subtillis* B7 as the original strain for the following domestication induction experiment to improve its conversion capability (Fig. 1).

**Figure 2:**
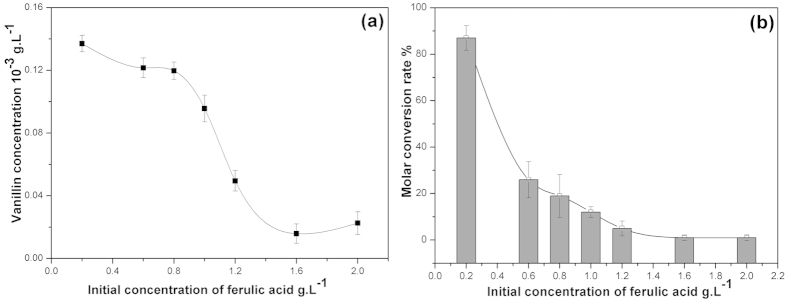
Ferulic acid resistance and conversion rate under different initial ferulic acid concentrations. (**a**) Effect of the initial ferulic acid concentration on the bioconversion of ferulic acid by *B. subtilis* B7-S (**b**) Efficiency of bioconversion by *B. subtilis* B7-S at different initial concentrations of ferulic acid.

Ferulic acid and vanillin both have an inhibitory effect on bacteria growth. Although *B. subtillis* could acquire energy from the medium to convert ferulic acid into vanillin, its growth is affected by the substrate concentration. The bacteria could grow and convert ferulic acid to vanillin when the ferulic acid concentration was between 0.2–0.8 g/L (Fig. 2(a)). However, when the ferulic acid concentration was higher than 0.8 g/L, the conversion capability was lower. When the ferulic acid concentration was 1.3 g/L, the rate of conversion by *B. subtillis* reached the lower limit. The bacteria could not grow on a medium with this high concentration of ferulic acid. To increase the tolerance concentration of the strains to ferulic acid, we chose an initial concentration of ferulic acid of 0.8–1.0 g/L. At this concentration, the production of vanillin by the bacteria began to decrease. Figure 2(b) shows the conversion rate at different concentrations of ferulic acid. When the ferulic acid concentration was low (0.2 g/L ferulic acid), the conversion rate was quite high; however, with the increase of the concentration of ferulic acid, the conversion rate by the bacteria was reduced. When the concentration reached 1.2 g/L, the conversion rate was lower than 5%. When it reached 1.6 g/L, the conversion rate was lower than 1%.

**Figure 3:**
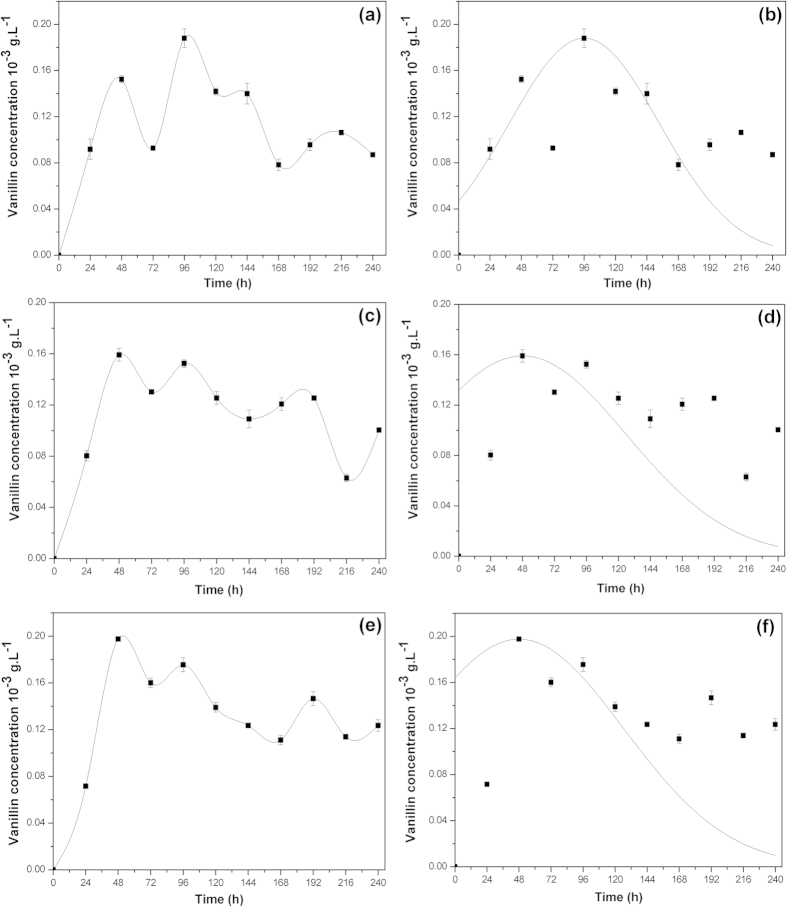
Adaptation of *Bacillus subtilis* B7-S to improve tolerance to ferulic acid. (**a**) Bioconversion of 0.8 g/L ferulic acid by *B. subtilis* B7-S (**b**) A test for the nonlinear model using 0.8 g/L ferulic acid (**c**) Bioconversion of 0.9 g/L ferulic acid by *B. subtilis* B7-S (**d**) A test for the nonlinear model using 0.9 g/L ferulic acid (**e**) Bioconversion of 1.0 g/L ferulic acid by *B. subtilis* B7-S (**f**) A test for the nonlinear model using 1.0 g/L ferulic acid.

When the ferulic acid concentration was 0.8 g/L, during the continuous passage of *B. subtillis* B7, the vanillin concentration reached a peak at 96 h (Fig. 3(a)). The experimental data were analyzed by the Gaussian peak function, and the results showed that when the ferulic acid concentration was 0.8 g/L, it took 96 hours to reach to the peak concentration of vanillin, which was 0.188 g/L (Fig. 3(b)). While the ferulic acid concentration was 0.9 g/L, it took 48 hours to reach the peak concentration of vanillin, which was 0.159 g/L (Fig. 3(d)). Compared to the process with a ferulic acid concentration of 0.8 g/L, the conversion process was 48 hours shorter. When the ferulic acid concentration was 1.0 g/L, it took 48 hours to reach the peak concentration of vanillin (Fig. 3(e)). However, Fig. 3(f) shows that when the initial ferulic acid concentration was 0.8 g/L, the peak concentration of vanillin was 0.198 g/L, which is higher 105.2% than the initial production.

**Figure 4:**
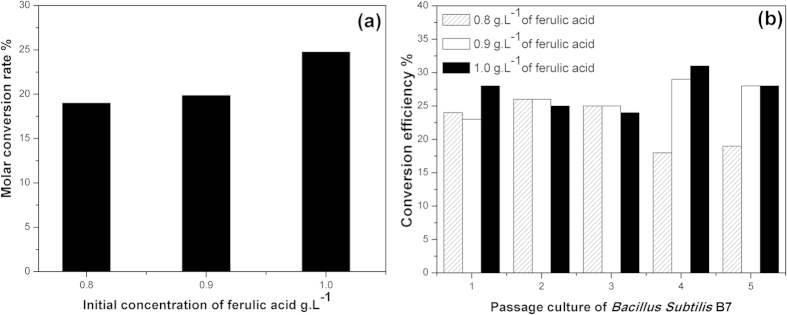
Effect of the initial concentration of ferulic acid on the bioconversion of ferulic acid by *B. subtilis* B7-S. (**a**) Conversion rate of 0.8–1.0 g/L ferulic acid by *B. subtilis* B7-S (**b**) Efficiency of bioconversion by *B. subtilis* B7-S at different initial concentrations of ferulic acid.

When the initial ferulic acid concentrations were 0.8 and 0.9 g/L, the conversion rates were 19% and 19.86%, respectively (Fig. 4(a)). While the initial ferulic acid concentration was 1.0 g/L, the molar conversion rate was 24.75%. This result shows that tolerance to ferulic acid by *B. subtillis* was enhanced, and the conversion capability was improved. To further analyze the changes in the conversion rate, we carried out a conversion experiment with the continuous passage of 5 generations, and the results are shown in Fig. 4(b). When the initial ferulic acid concentration was 0.8, 0.9, or 1.0 g/L, during the first 3 generations of conversion process, the molar efficiency was almost the same, ranging from 23% to 28% (Fig. 4(b)). However, in the 4^th^ generation of the conversion experiment, the conversion efficiency at a ferulic acid concentration of 0.8 g/L decreased to 18%. Meanwhile, the groups with ferulic acid concentrations of 0.9 g/L and 1.0 g/L had conversion efficiencies of 29% and 31%, respectively. Based on the results shown in Fig. 4(a,b), the molar conversion rate increased with an increase in the ferulic acid concentration, which indicates that the conversion capability of the bacteria was enhanced. Meanwhile, at higher substrate concentrations, there were higher efficiencies of conversion, which indicates that the tolerance to ferulic acid by the strains was enhanced. The tolerance capability to ferulic acid by *B. subtillis* B7 is due to adaptation of the strain, which also results in the enhancement of ferulic acid conversion under these environmental stress conditions.

**Figure 5:**
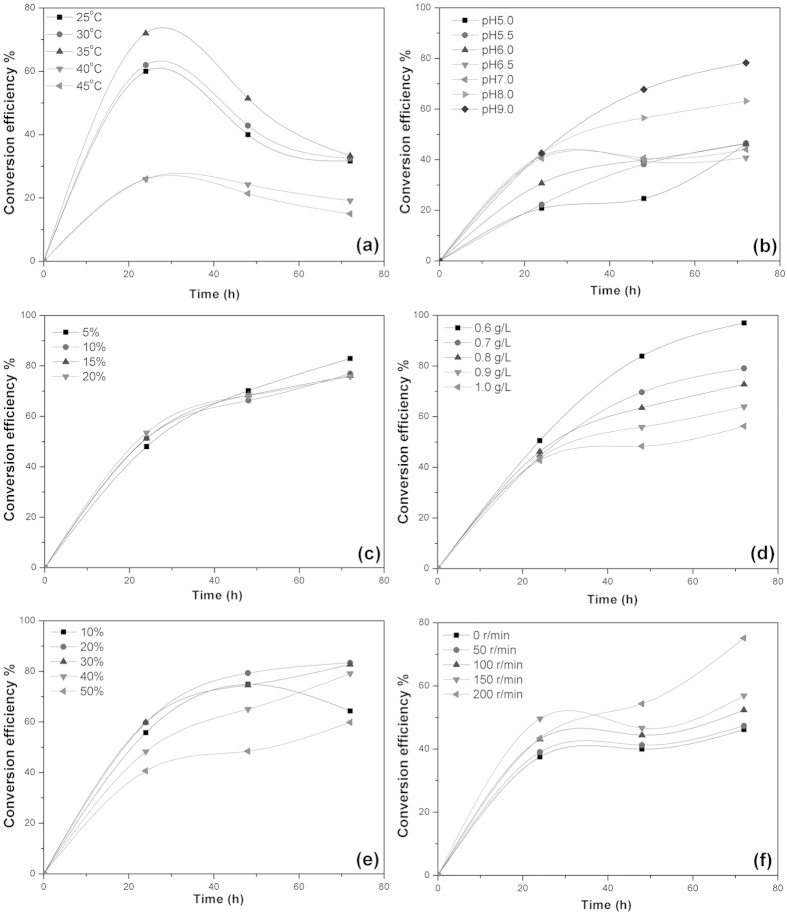
Effect of different factors on the bioconversion of ferulic acid by *B. subtilis* B7-S. (**a**) Effect of temperature on the bioconversion of ferulic acid by *B. subtilis* B7-S (**b**) Effect of initial pH on the bioconversion of ferulic acid by *B. subtilis* B7-S (**c**) Effect of inoculum volume on the bioconversion of ferulic acid by *B. subtilis* B7-S (**d**) Effect of substrate concentration on the bioconversion of ferulic acid by *B. subtilis* B7-S (**e**) Effect of volume of culture medium on the bioconversion of ferulic acid by *B. subtilis* B7-S (**f**) Effect of shaking speed on the bioconversion of ferulic acid by *B. subtilis* B7-S.

Figure 5(a) shows the effect of temperature on the bioconversion of ferulic acid by *B. subtilis* B7-S at five different temperatures (25, 30, 35, 40 and 45 °C). As shown in the figure, the conversion efficiency did not vary significantly over a temperature range of 25–30 °C, whereas it decreased significantly when the temperature was above 35 °C. The maximum conversion efficiency was 72% at 35 °C. The results indicated that the temperature might be an important factor for the energy-dependent mechanisms in ferulic acid bioconversion by microbial cells.

It is well known that pH plays a crucial role in microbial growth and bioconversion processes. Generally, variations in pH can strongly influence the chemistry of the substrate, regulate the activity of functional groups on the microbial cell walls, and alter enzymes involved in the conversion reaction^26^. The effect of the initial pH on the bioconversion of ferulic acid by *B. subtilis* B7-S is graphically presented in Fig. 5(b). As shown in the figure, the conversion efficiency did not vary significantly over a pH range of 5.0–7.0. However, the conversion efficiency increased significantly when the pH was above 7.0, and it attained a maximum value of 78.33% at pH 9.0. These results imply that the optimal growth of *B. subtilis* B7-S and the bioconversion of ferulic acid occur at alkaline pH values. Similar results were observed in a previous report^27^. The influence of the inoculum size on the bioconversion of ferulic acid by *B. subtilis* B7-S was investigated by using different inoculum volumes in the range of 5–20% in Fig. 5(c). Therefore, considering the expense of inoculum preparation, 5% was chosen as the optimum inoculation volume.

The effects of the initial ferulic acid concentration on the bioconversion are shown in Fig. 5(d). The results showed that with the increase of the initial concentration from 0.6 to 1.0 g/L, the concentrations of vanillin in the cultures increased immediately. However, the conversion efficiency decreased from 98.7 to 55.48%. The decrease in the bioconversion efficiency with the increase of the initial concentration of ferulic acid may be attributed to a lack of available microbial cells to accommodate additional ferulic acid in the solution. Ferulic acid was reported to be toxic to microorganisms, and it may damage intracellular hydrophobic sites and cause ion leakage by altering membrane permeability, leading to cell death at high concentrations^28^. Moreover, vanillin is also toxic to many living organisms. Therefore, in the case of a lower initial concentration, the substrate has no effect on microorganisms, and the entire amount of ferulic acid could interact with the microbial cells, resulting in a higher bioconversion efficiency. With the increase of the initial concentration, the high concentrations of ferulic acid and vanillin will have negative effects on the organism, resulting in a lower bioconversion efficiency.

Oxygen concentration, one of the most important factors, not only influences the growth of *B. subtilis* B7-S, but it also affects the bioconversion of ferulic acid. The effects of oxygen concentration on the bioconversion of ferulic acid by *B. subtilis* B7-S were investigated by using different percent volume of culture medium from 10–60%, as shown in Fig. 5(e), and various shaking speeds, as shown in in Fig. 5(f). The conversion efficiency decreased with increases in volume of culture medium, and the maximum conversion efficiency was 83.4% when the volume of culture medium was 20%, as shown in Fig. 5(e). Furthermore, the conversion efficiency increased with increasing shaking speed, and the maximum conversion efficiency was 75.13% when the shaking speed was 200 r/min, as shown in Fig. 5(f). The lower the volume of culture medium or the higher the shaking speed, the higher the oxygen concentrations. The high oxygen concentration promotes the growth of microbial cells and it can also improve the bioconversion. Therefore, according to the above analysis, the optimum conversion conditions for the production of vanillin with *B. subtilis* B7-S may be summarized as follows: temperature, 35 °C; initial pH, 9.0; inoculum volume, 5%; ferulic acid concentration, 0.6 g/L; volume of culture medium, 20%; and shaking speed, 200 r/min.

**Figure 6:**
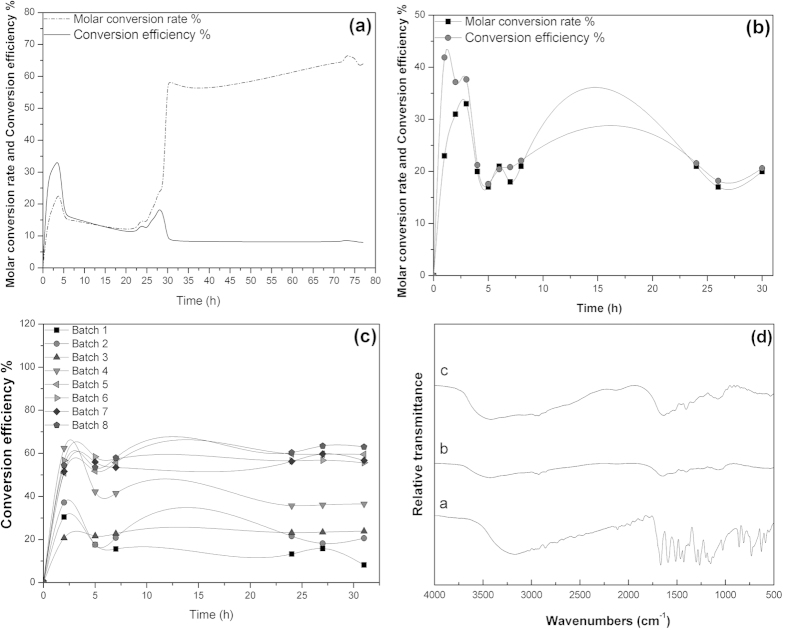
Results for different fermentation batches. (**a**) Results of the first fermentation experiment (**b**) Results of the second fermentation experiment (**c**) Results of 1^st^ through the 8^th^ fermentation batches (**d**) FT-IR spectra of the crude and standard vanillin (**a**: standard vanillin; **b,c**: treated vanillin).

Based on the results of shake-flask fermentation, repeated batch fermentation was carried out in a 5-L fermenter. Suspensions of *B. subtilis* B7-S were used as inoculums, and the medium and operating conditions were as described in Section 2.3 and Section 2.4, respectively. The molar conversion ratio and conversion efficiency were simultaneously determined for the period (approximately 1 h) of the first fermentation. As shown in the figure, the maximum molar conversion was achieved within 30 h (65%), and thereafter, the conversion efficiency decreased to a value below 9% as the fermentation time increased, as shown in Fig. 6(a). This result was attributed to the high density of biomass and the low conversion efficiency. When the maximum vanillin concentration was detected, the fermented broth was replaced. Therefore, a second fermentation experiment was carried out over a 30-h period. The molar conversion ratio and conversion efficiency attained a maximum value after 3 h of fermentation, and thereafter, they did not show any significant change, as presented in Fig. 6(b).

To further investigate the stability of the fermentation process, eight small-scale fermentation experiments were performed. As shown in Fig. 6(c), the conversion efficiency changed significantly and the value increased from 8.19% to 36.48% from the 1^st^ to the 4^th^ fermentation batch. However, the conversion efficiency tended to stabilize between 55% and 63%, thus attaining the current rate of bioconversion using microorganisms in industrial applications.

The FT-IR spectra of the standard and crude vanillin were taken to investigate the nature of the bioconversion product (Fig. 6(d)). The FT-IR peaks at 3171, 2924, 2475, 1667, 1591, 1372, 1152, 1026, 859, and 629 cm^−1 ^of the standard vanillin corresponded to the -OH, -NH, -CH, amide I, and amide II bands of protein peptide bonds, the -COOH groups, and the -CN stretching, C=O stretching and -CN stretching vibrations. The IR spectrum of sample c was closer to sample b. The primary reason is that sample b dried for 3 days, while sample c dried for 6 days. These FT-IR spectra can be used as a quality standard to assess the vanillin produced by bioconversion.

More recently, in EU Commission Regulation (EC) No. 629/2008^29^, the maximum level of lead for all food supplements was set at 3.0 mg/kg. Although no maximum level has yet been established in Europe for arsenic, the report on the exposure of the European population to heavy metals in the diet showed that the concentration of arsenic is generally less than 0.25 mg/kg^29^. The ICP-AES analysis indicated that the concentrations of lead and arsenic in crude vanillin were both less than 0.018 mg/kg. These results implied that the vanillin produced from ferulic acid via bioconversion is safe.

**Figure 7:**
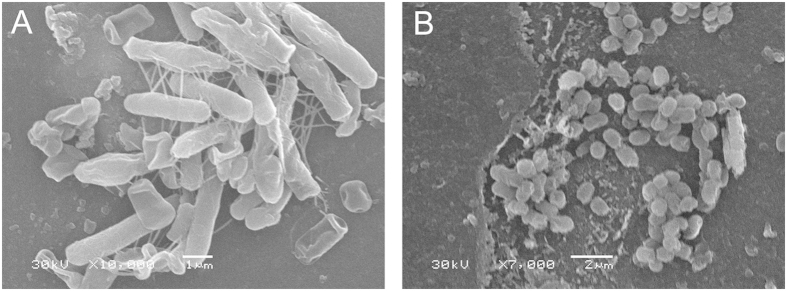
An SEM image of the surface of *B. subtilis* B7. (**A**) Unadapted *Bacillus subtilis* B7 before bioconversion (scale bar = 1 μm) (**B**) The adaptation of *Bacillus subtilis* B7 after bioconversion (scale bar = 2 μm).

**Figure 8:**
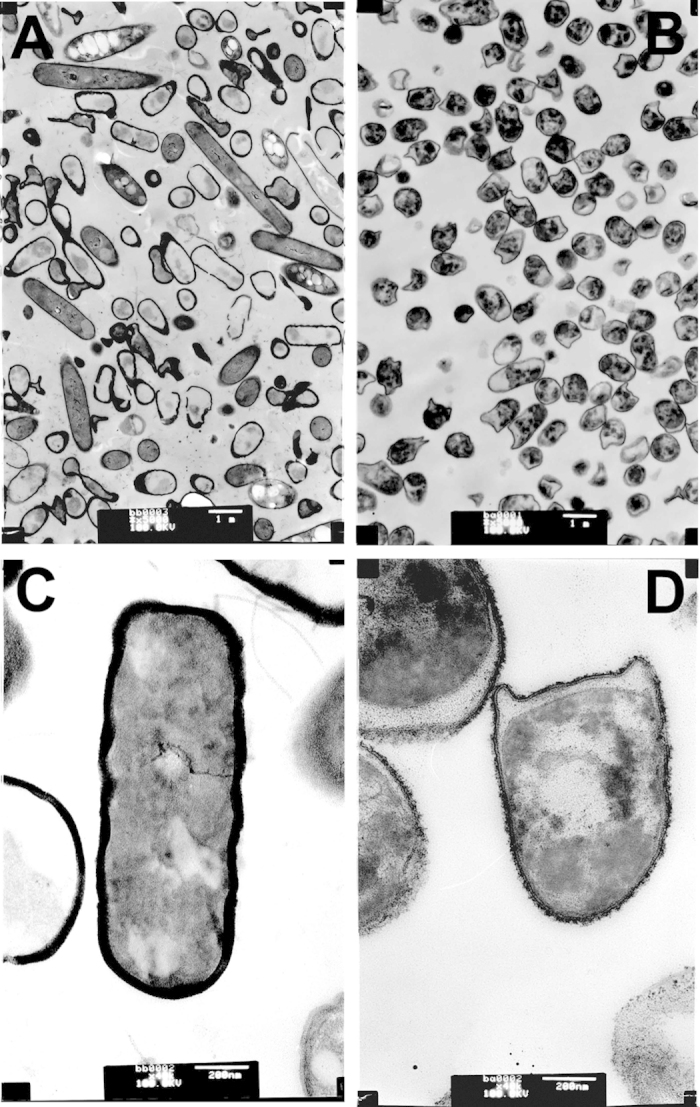
An TEM image of the surface of *B. subtilis* B7. (**A**,**C**) Unadapted *Bacillus subtilis* B7 before bioconversion (**B,D**) The adaptation of *Bacillus subtilis* B7 after bioconversion (**A,B**) scale bar = 1 μm; (**C,D**) scale bar = 200 nm.

The SEM analysis of the original strain and the domestic strain as well as the original strain, non-domestic strain and the induced wild strain (Fig. 7(a)). The bacteria cell is rodlike, and the size of the cell is 4.0–6.0 μm. Figure 7(b) shows domestic *B. subtillis* B7-S, which is significantly different in cell morphology, changing from a rodlike morphology to a sphere, which is relevant to the adaptation to the environment. At higher concentrations of ferulic acid, the bacteria became spherical to reduce the area in contact with the ferulic acid. Figure 8 shows the TEM analysis of the original strain and the domestic strain. Figure 8(A,C) show the original B7-S strain, the non-domestic strain and the induced wild strain. The bacteria are rodlike with a thicker cell wall and flagella outside the cell. Figure 8(B,D) show domestic *B. subtillis* B7-S, which has an irregular spherical morphology, a thinner cell wall, a rough external cell surface, reduced cytoplasm, and cytosol close to the side of the cell wall; the flagella could not be detected. Thus, the cell morphology was altered to adapt to the high concentration of ferulic acid. Furthermore, ferulic acid has an inhibitory effect on cell survival.

## Methods

### Microorganism strains and culturing conditions

Ferulic acid and 2-thiobarbituric acid were purchased from Qin Chemical Reagent Co., Ltd. (Shanghai, China). Vanillin was purchased from Sigma–Aldrich and Tianxin Chemical Reagent Co., Ltd. (Tianjin, China). All other chemicals were analytical grade. Several bacteria, yeast and fungi can employ ferulic acid as substrate to produce vanillin. The strains screened are listed in Table 1. *B. subtilis* B7-S (CCTCC M2011162) was deposited in the China Center for Type Culture Collection (CCTCC).

### Nucleotide sequence accession number

The draft sequence of *B. subtilis* B7-S under this whole-genome shotgun project has been deposited at DDBJ/EMBL/GenBank under the accession no. AZNI00000000^30^. The full length of 16S rRNA gene of *B. subtilis* B7-S are deposited at GenBank with an accession number JQ086379.

### Optimization of bioconversion of ferulic acid

All batch experiments were carried out in Erlenmeyer flasks on a horizontal water-bath shaker. To investigate the influence of temperature, initial pH, inoculum volume, substrate concentration, volume of culture medium and shaking speed on the bioconversion of ferulic acid by *B. subtilis* B7-S, different conditions of temperature (25–45 °C), pH (5.0–9.0), inoculum volume (5–20%), substrate concentration (0.6–1.0 g/L), volume of culture medium (10–60%) and shaking speed (0-200 r/min) were evaluated in this study. The pH of the solutions was adjusted by adding 1.0 M HCl or 1.0 M NaOH.

### Small-scale bioconversion experiments

The small-scale bioconversion experiments was carried out in a 5-L fermenter with a 3-L working volume with a 5% inoculum volume at an incubation temperature of 35 °C, an agitation speed of 200 r/min, an aeration rate of 0.5 L/min and 0.1 MPa pressure. Time-course samples for the batch experiments and small-scale bioconversion experiments were all taken and analyzed. All experiments were conducted in triplicate, and the average values were reported.

### Analytical procedures

The vanillin content was determined by the colorimetric thiobarbituric acid assay with the modifications^31^. The cell concentration was determined in terms of the optical dispersion of the culture samples at a wavelength of 600 nm using a UV–Vis Spectrophotometer. The metal content in the supernatant liquid was analyzed by an inductively coupled plasma atomic emission spectrometer (ICP-AES, IRIS Advantage ER/S, Thermo Jarrell Ash, USA). FT-IR spectroscopy was used to characterize the bioconversion products based on the procedure of Yan *et al.*^32^. The microscopic features of the samples were observed through JEOL scanning electron microscope (SEM; JSM-5600LV, Tokyo, Japan) operated at 20 kV and JEOL transmission electron spectroscopy (TEM; JEM-1230, Japan) operated at 100 kV. The sample preparation method for SEM analysis was previously described^33^.

The molar conversion ratio (molar yield) of vanillin was obtained by Equation (1), and the conversion efficiency of vanillin was calculated by Equation (2). The data were analyzed using a non-linear curve fitting analysis with the Amplitude version of the Gaussian peak function in the Origin Pro 8.0 package (OriginLab, USA). The Gaussian peak model is shown in Equation (3).


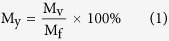



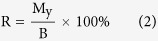



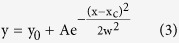


where M_y_ and R are the molar conversion ratio (%) and conversion efficiency (%), respectively, M_v_ and M_f_ are the molar number of vanillin and ferulic acid in medium, respectively, and B is the cell concentration (OD_600nm_). where A is the amplitude (peak height), w is the peak width of A_1/2_, and X_c_ is the center of the peak value, respectively.

## Additional Information

**How to cite this article**: Chen, P. *et al.* A microbial transformation using *Bacillus subtilis* B7-S to produce natural vanillin from ferulic acid. *Sci. Rep.* **6**, 20400; doi: 10.1038/srep20400 (2016).

## Figures and Tables

**Table 1:** Microbial conversion of ferulic acid by various microorganisms.

Microorganism	GenBank Accession numbers	Molar conversion rate (%)	Conversion efficiency (%)
*Bacillus subtilis* B7	JQ086378	13.33%	42.45%
*Bacillus subtilis* B7-S	JQ086379	18.42%	63.30%
*Bacillus stearothermophilus*	ND a	3.59%	12.61%
*Enterobacter cloacae* Y219	JQ086381	9.59%	29.25%
*Bacillus coagulans*	ND	7.25%	24.74%
*Enterobacter aerogenes*	ND	3.79%	10.97%
*Corynebacterium glutamicum*	ND	2.21%	10.27%
*Pseudomonas putida*	ND	4.48%	12.44%
*Escherichia coli* BL21	ND	2.55%	16.17%
*Candida Kefyr*	ND	1.69%	3.99%
*Kluyveromyces lactis* B9	JQ086382	1.37%	3.86%
*Rhodotorula lactosa* C8	JQ086383	2.46%	7.08%
*Kluyveromyces marxianus* D10	JQ086384	2.59%	7.64%
*Kluyveromyces marxianus* 15D	JQ086385	1.24%	5.08%
*Rhodotorula rubra*	ND	1.18%	7.57%
*Streptomyces thermophila*	ND	5.90%	16.72%
*Boletus edulis* KX920NG	JQ086386	0.99%	4.92%
*Cantharellus sp.* KX560JY	JQ086387	0.92%	7.45%
*Artomyces pyxidatus* KX320SH	JQ086388	0.92%	9.06%

^a^ND, no detection.
